# Comparison of measurement methods with a mixed effects procedure accounting for replicated evaluations (COM_3_PARE): method comparison algorithm implementation for head and neck IGRT positional verification

**DOI:** 10.1186/s12880-015-0074-z

**Published:** 2015-08-28

**Authors:** Anuradha Roy, Clifton D. Fuller, David I. Rosenthal, Charles R. Thomas Jr.

**Affiliations:** Department of Management Science and Statistics, The University of Texas at San Antonio, One UTSA Circle, San Antonio, 78249 TX USA; Department of Radiation Oncology, The University of Texas M.D. Anderson Cancer Center, Houston, TX USA; Department of Radiation Medicine, Oregon Health & Science University, Portland, OR USA

## Abstract

**Purpose:**

Comparison of imaging measurement devices in the absence of a gold-standard comparator remains a vexing problem; especially in scenarios where multiple, non-paired, replicated measurements occur, as in image-guided radiotherapy (IGRT). As the number of commercially available IGRT presents a challenge to determine whether different IGRT methods may be used interchangeably, an unmet need conceptually parsimonious and statistically robust method to evaluate the agreement between two methods with replicated observations. Consequently, we sought to determine, using an previously reported head and neck positional verification dataset, the feasibility and utility of a Comparison of Measurement Methods with the Mixed Effects Procedure Accounting for Replicated Evaluations (COM_3_PARE), a unified conceptual schema and analytic algorithm based upon Roy’s linear mixed effects (LME) model with Kronecker product covariance structure in a doubly multivariate set-up, for IGRT method comparison.

**Methods:**

An anonymized dataset consisting of 100 paired coordinate (X/ measurements from a sequential series of head and neck cancer patients imaged near-simultaneously with cone beam CT (CBCT) and kilovoltage X-ray (KVX) imaging was used for model implementation. Software-suggested CBCT and KVX shifts for the lateral (X), vertical (Y) and longitudinal (Z) dimensions were evaluated for bias, inter-method (between-subject variation), intra-method (within-subject variation), and overall agreement using with a script implementing COM_3_PARE with the MIXED procedure of the statistical software package SAS (SAS Institute, Cary, NC, USA).

**Results:**

COM_3_PARE showed statistically significant bias agreement and difference in inter-method between CBCT and KVX was observed in the Z-axis (both *p* − value<0.01). Intra-method and overall agreement differences were noted as statistically significant for both the X- and Z-axes (all *p* − value<0.01). Using pre-specified criteria, based on intra-method agreement, CBCT was deemed preferable for X-axis positional verification, with KVX preferred for superoinferior alignment.

**Conclusions:**

The COM_3_PARE methodology was validated as feasible and useful in this pilot head and neck cancer positional verification dataset. COM_3_PARE represents a flexible and robust standardized analytic methodology for IGRT comparison. The implemented SAS script is included to encourage other groups to implement COM_3_PARE in other anatomic sites or IGRT platforms.

## Background

Method comparison is a frequent problem encountered whenever different measurement devices/techniques are implemented in the absence of a gold standard [1–7]. Method comparison in radiological science is often a vexing issue [8–15], and is especially notable when competing imaging methodologies are used without establishment of the technical superiority in terms of accuracy of one platform. In a specific example, the explosion in applications of image-guided radiation therapy (IGRT), which necessitates repeated and exceedingly accurate spatial localization in order to carefully deliver conformal radiation dose, places a premium on both reproducibility and accuracy [14, 16–25]. Furthermore, the large number of divergent manufacturer-supported mechanisms for achieving image-guided target localization/positional verification (e.g., 2D- and 3D-ultrasound [26–34], 2-D radiography [35–39], megavoltage and kilovoltage 3-D tomography [40–44]) have arisen in the absence of a gold-standard, and thus have been the impetus for a large number of inter-modality comparative studies, which themselves often utilize a wide array of statistical methods to report between method measurement differences [22, 24, 25, 27, 33, 34, 44–47]. In an effort to more formally assess both inter- and intra-method bias, as well as to streamline comparatively time- and effort-intensive graphical and statistical analysis inherent in many method comparison statistical techniques, we sought to devise an algorithm to explore agreement between two methods of image-guided radiotherapy, using a novel linear mixed effects (LME) model with Kronecker product covariance structure in a doubly multivariate approach [48]. This integrated approach has great potential utility, formally evaluating inter-method bias, inter-subject variability and the intra-subject variability (i.e., agreement between the repeatability coefficients) of the two imaging methods/devices. Testing of all three aspects is crucial, as inter-subject variability is of import when estimating the difference between the two methods giving different measurements on the same subject, while intra-subject variability affords calculation of the random error among the replications taken by the same method on the same subject [49]. We use a doubly multivariate set-up (i.e., measurement data for each subject is considered at two levels, incorporating both the number of measurement methods and number of replicated measurements). This specific LME-based technique, which we shall refer to as COM_3_PARE (Comparison of Measurement Methods with Mixed Effects Procedure Accounting for Replicated Evaluations) is robust with regard to number of replicates, and is easily performed using SAS software (*vide infra*). LME models have improved fidelity in scenarios wherein observations are not fully independent and can more correctly models correlated errors, compared to general linear models (GLM), which includes typical statistical analyses (e.g., t-test, ANOVA, linear regression). LME includes multiple random effect components, compared to single element evaluation in most GLM models, affording improved analysis of continuous variables where random effects, multiple hierarchical data levels, and replicated measurements are concerned. The number of replicated measurements on each patient or subject may not be equal, and also the number of replications of the two methods on the same subject may not be equal. The specific aims for this study included:

- First known application of LME-based COM_3_PARE hypothesis testing protocol for method comparison using imaging data.
- Demonstration of feasibility and utility of COM_3_PARE using an established head and neck positional verification dataset, previously presented with standard method comparison approaches.

## Methods

### Datasets

A previously presented dataset consisting of a series of 100 paired measures using two distinct positional verification techniques in a series of 28 sequential head and neck squamous cell carcinoma patients was utilized. As this manuscript is designed to specify a novel statistical methodology, interested readers are referred to the previous manuscript [50], wherein imaging parameters have been previously detailed. Briefly, CBCT and stereoscopic kV X-ray were acquired near-simultaneously at approximately biweekly intervals throughout a patient’s course of treatment (dependent upon the scheduling exigencies in the department) for a series of patients with head and neck cancers. CBCT/kV X-ray analyses were performed using the attached on-board imager (Varian Medical Systems, Palo Alto, CA). Positional verification was performed with manufacturer-supplied software (Varian OBI 1.3/Varian Vision, Varian Medical Systems, Palo Alto, CA) for 3D-3D (CBCT-simulation CT) and 2D-2D (kV X-ray-DRR) automated matching using the aforementioned software. Recorded shifts represent the coregistration/allineation software derived values without physician/observer modification. For each paired-method positional acquisition, the origin was defined as the point in space identified by the initial isocenter position using immobilization-mask-based markers. Utilizing a three-dimensional Cartesian coordinate system, this spatial location was designated as a ‘zero point’ with X/Y/Z coordinates of (0, 0, 0). Software-derived shifts for each system were recorded in centimeters, specified as X-, Y- or Z-axis. Software- derived shifts were characterized as X- (lateral or left/right), Y- (vertical or anteroposterior) and Z- (longitudinal or superoinferior) axes, respectively, for both kV X-ray and CBCT IGRT techniques. For the purposes of clarity, we proposed the following three conditions be met to verify whether two methods for measuring a variable (in this specific case, IGRT-suggested spatial shifts) can be considered interchangeable:

1. No significant bias (i.e., no difference between the means of the two methods under a pre-specified threshold nor a statistically significant difference between said means).
2. No statistically significant difference in the inter-subject (between-subject) variability of the two methods.
3. No statistically significant difference in the intra-subject (within-subject) variability (i.e., repeatability) of the two methods.

For this study, we pre-specified a bias threshold of an absolute value of <0.1 cm, with a statistically significant difference designated by *α*<0.05. To assess the aforementioned criteria, we implemented the LME methodology proposed by Roy^48^, referred to as COM_3_PARE (see Appendix A).

### Statistical analysis with COM_3_PARE

As mentioned in the introduction the number of replicated measurements on each patient or subject may not be equal, and also the number of replications of the two methods on the same subject may not be equal. Let $p^{KVX}_{i}$ and $p^{CBCT}_{i}$ be the number of replications on subject *i* by the established method (KVX), and a new method (CBCT) respectively. Let $p_{i}= \max \left ({p^{KVX}_{i}, p^{CBCT}_{i}}\right)$, and *n*_*i*_=2*p*_*i*_. Therefore, the number of observations on the *i*th subject is *n*_*i*_, under the assumption that the *i*th subject has $\left |p^{KVX}_{i}- p^{CBCT}_{i}\right |$ missing values.

Let $y^{KVX}_{\textit {it}}$ and $y^{CBCT}_{\textit {it}}$ be the responses by the established method and a new method of the *i*th subject at the *t*th replicate, *i*=1,2,…,*N*, *t*=1,2,…,*p*_*i*_. Let $\boldsymbol {y}_{\textit {it}}= \left (y^{KVX}_{\textit {it}}, y^{CBCT}_{\textit {it}}\right)^{\prime }$ be the 2×1 vector of measurements corresponding to the *i*th subject at the *t*th replicate. Let $\boldsymbol {y}_{i} = \left (\boldsymbol {y}_{i1}^{\prime }, \boldsymbol {y}_{i2}^{\prime }, \ldots, \boldsymbol {y}_{{ip}_{i}}^{\prime }\right)^{\prime }$ be the (*n*_*i*_×1)-dimensional random vector corresponding to the *i*th subject. That is, the vector ***y***_*i*_ is obtained by stacking the responses of the KVX method, and the CBCT method at the first replication, then stacking the responses of the KVX method and the CBCT method at the second replication and so on. We write all responses (***y***_*i*_) of the *i*th subject in a matrix equation as

$$\begin{array}{@{}rcl@{}} \quad \quad \;\;\; \boldsymbol{y}_{i} &=& \boldsymbol{X}_{i} \boldsymbol{\beta} + \boldsymbol{Z}_{i} \boldsymbol{b}_{i} + \boldsymbol{\epsilon}_{i}, \\ \text{with} \quad \boldsymbol{b}_{i} &\sim& N_{m}(\boldsymbol{0}, \boldsymbol{D}),\\ \text{and} \quad \; \boldsymbol{\epsilon}_{i} &\sim& N_{n_{i}}(\boldsymbol{0}, \boldsymbol{R}_{i}), \end{array} $$

where ***b***_1_,***b***_2_,…,***b***_*N*_,***ε***_1_,***ε***_2_,…,***ε***_*N*_ are independent, and ***y***_1_,***y***_2_,…,***y***_*N*_ are also all independent. LME model allows for the explicit analysis of between-subject (***D***) and within-subject (***R***_*i*_) sources of variation of the two methods. We define the two methods by a vector variable Mvar; Mvar=1 for the KVX method and Mvar=2 for the CBCT method. We choose the intercept and the vector variable Mvar as fixed effects, thus the design matrix ***X***_*i*_ has three columns, and consequently ***β***=(*β*_*o*_,*β*_1_,*β*_2_)^′^ is a 3-dimensional vector containing the fixed effects. We also choose the vector variable Mvar as random effects, i.e., Mvar is random across individual subjects; thus the design matrix ***Z***_*i*_ has two columns. Therefore, ***b***_*i*_= (*b*_1*i*_,*b*_2*i*_)^′^ is a 2-dimensional vector containing the random effects.

The solution for ***β*** gives the means of the two methods *μ*_*KVX*_ and *μ*_*CBCT*_. The between-subject variance-covariance matrix ***D*** of the KVX method and the CBCT method is a general (2×2)-dimensional matrix, and ***R***_*i*_ is a (*n*_*i*_×*n*_*i*_)-dimensional covariance matrix which depends on *i* only through its dimension *n*_*i*_. The marginal density function of $\boldsymbol {y}_{i} \sim N_{n_{i}}(\boldsymbol {X_{i}} \boldsymbol {\beta }, \boldsymbol {Z}_{i} \boldsymbol {D} \boldsymbol {Z}_{i}^{\prime } + \boldsymbol {R}_{i})$. Suppose the matrix ***Σ*** represents the within-subject variance-covariance matrix of the KVX method and the CBCT at any replicate; also, suppose ***V*** represents the *p*×*p*-dimensional correlation matrix of the replicated measurements on a given method, where $p=\underset {{i}}{\text {max}}(p_{i})$. It is assumed that the 2×2 within-subject variance-covariance matrix ***Σ*** is same for all replications, and the correlation matrix ***V*** is assumed to be the same for both the methods. We assume $\boldsymbol {R}_{i} =\underset {n_{i}}{\text {dim}} (\boldsymbol {V}\otimes \boldsymbol {\Sigma })$, where ***V*** and ***Σ*** respectively are positive definite matrices as described above, and ⊗ represents the Kronecker product structure. The notation $\underset {n_{i}}{\text {dim}} (\boldsymbol {V}\otimes \boldsymbol {\Sigma })$, represents a (*n*_*i*_×*n*_*i*_)-dimensional submatrix obtained from the (2*p*×2*p*)-dimensional matrix (***V***⊗***Σ***), by appropriately keeping the columns and rows corresponding to the *n*_*i*_-dimensional response vector ***y***_*i*_. Since the equicorrelated or compound symmetry (CS) structure assumes equal correlation among all replicated measurements, we assume that the correlation matrix ***V*** of the replicated measurements has equicorrelated correlation structure. For the above design matrix ***Z***_*i*_ and between-subject ***D*** and within-subject ***R***_*i*_ sources of variation, the observed (*n*_*i*_×*n*_*i*_)-dimensional overall variance-covariance matrix ***Ω***_*i*_ for the *i*th individual is given by

$$\begin{array}{@{}rcl@{}} \text{Cov}(\boldsymbol{y}_{i})= \boldsymbol{\Omega}_{i} &=&\boldsymbol{Z}_{i} \boldsymbol{D} \boldsymbol{Z}_{i}^{\prime} + \boldsymbol{R}_{i},\\ &=& \boldsymbol{Z}_{i} \boldsymbol{D} \boldsymbol{Z}_{i}^{\prime} + \underset{n_{i}}{\text{dim}} (\boldsymbol{V}\otimes \boldsymbol{\Sigma}). \end{array} $$

Thus, the covariance matrix has the same structure for each subject, except that of the dimension. The 2×2 block diagonals Block ***Ω***_*i*_ in the overall variance-covariance matrix ***Ω***_*i*_ represent the overall variance-covariance matrix between the two methods. Similarly, the 2×2 block diagonals in the overall correlation matrix ***Ω***_*i*__Correlation represent the overall correlation matrix between the two methods. Thus, the off-diagonal element in this 2×2 overall correlation matrix gives the overall correlation between the two methods. It can be easily seen that the overall variability is the sum of between-subject variability and within-subject variability (see Roy^48^ for detail). Thus, we see that if there is a disagreement in overall variabilities, then it may be due to the disagreement in either between-subject variabilities or within-subject variabilities or both.

## MIXED procedure of SAS

We use MIXED procedure (*PROC MIXED*) of *SAS* to get the maximum likelihood estimates (MLEs) of ***β***,***D***, ***R***_*i*_ and ***Ω***_*i*_. *METHOD=ML* specifies MIXED procedure to calculate the maximum likelihood estimates of the parameters. The COVTEST option requests hypothesis tests for the random effects. *CLASS* statement specifies the categorical variables. *DDFM=KR* specifies the Kenward-Roger^51^ correction for computing the denominator degrees of freedom for the fixed effects. Kenward-Roger correction is suggested whenever one has replicated or repeated measures data; also for missing data. The *SOLUTION (S)* option in the *MODEL* statement provides the estimate of the difference between the two mean readings (bias) of the two methods. *RANDOM* and *REPEATED* statements specify the structure of the covariance matrices ***D*** and ***R***_*i*_. See the sample program in Appendix A that demonstrates the use of *RANDOM* and *REPEATED* statements. *PROC MIXED* calculates the (*n*_*i*_×*n*_*i*_)-dimensional submatrix ***R***_*i*_ of the *i*th subject from the (2*p*×2*p*)-dimensional matrix (***V***⊗***Σ***), and eventually calculates (*n*_*i*_×*n*_*i*_)-dimensional submatrix ***Ω***_*i*_. When the number of replications on each subject by respective methods is unequal, *PROC MIXED* considers the case as missing value situation. Options *V*=3 and *VCORR*=3 in the *RANDOM* statement give the estimate of the overall variance-covariance matrix ***Ω***_3_ and the corresponding ***Ω***_3__Correlation matrix, i.e., for the third subject. The option *G* in the *RANDOM* statement gives the estimate of the between-subject variance-covariance matrix ***D***. Option *R* in the *REPEATED* statement gives the estimate of the variance-covariance matrix ***R***_1_ for the first subject. One can get the ***Ω***_*i*_ variance-covariance matrix and the corresponding ***Ω***_*i*__Correlation matrix for all subjects by specifying *V*= 1 to N, and *VCORR*=1 to N in the *RANDOM* statement. When the correlation matrix ***V*** on the replicated measurements assumes equicorrelated structure and ***Σ*** as unstructured, we use the option *TYPE=UN* along with *SUBJECT=REPLICATE(PATIENT)* in the *REPEATED* statement. This gives the 2× 2 within-subject variance-covariance matrix ***Σ***. See Appendix A.

## Related hypotheses testings to test the disagreement between KVX and CBCT

If there is a disagreement between the two methods, it is important to know whether it is due to the bias, due to the difference in between-subject variabilities or due to the difference in within-subject variabilities of the two methods. If it is due to the bias between the two methods, it is easy to correct. The output of *PROC MIXED* always gives the bias, its *t* − value and its *p* − value. Nonetheless, it is not straightforward to check the agreement or disagreement in between-subject variabilities and in within-subject variabilities of the two methods. We will accomplish these by the indirect use of *PROC MIXED* in two steps (described below) by using likelihood ratio tests.

### Testing of hypothesis of difference between the means of KVX and CBCT

We are interested in testing the following hypothesis for bias:

$$\begin{array}{@{}rcl@{}} &&H_{\mu}: \text{the two methods do not have the same mean},  \\ \text{vs.} \;\; && K_{\mu}: \text{the two methods have the same mean}.  \end{array} $$

Output of *PROC MIXED* (Solution for Fixed Effects) gives the bias and the corresponding *t* − value and *p* − value.

### Testing of hypothesis of difference in between-subject variabilities of KVX and CBCT

Here we are interested in testing the following hypothesis:

$$\begin{array}{@{}rcl@{}} H_{d}:&& \text{the two methods do not have the same}\\ &&\text{between-subject variabilities},\\ \text{vs.}\;\;\;\;\; K_{d}:&& \text{the two methods have the same}\\ &&\text{between-subject variabilities}.  \end{array} $$

We apply the likelihood ratio test for this hypothesis testing. To compute the test statistic −2 ln*Λ*_*d*_, where

$$\begin{array}{@{}rcl@{}} \qquad \; \; -2 \ln \Lambda_{d} =\left[-2 \ln \max_{K_{d}} L \right] - \left[-2 \ln \max_{H_{d}} L\right]. \end{array} $$

The log likelihood function under both null hypothesis and alternating hypothesis must be maximized separately. We do this by setting the option *METHOD=ML* in *PROC MIXED* statement. The option *TYPE=UN* in the *RANDOM* statement, along with the option *TYPE=UN* in the *REPEATED* statement, is used to calculate the “-2 Log Likelihood" for the covariance structure under *H*_*d*_. Similarly, the option *TYPE=CS* in the *RANDOM* statement, along with the option *TYPE=UN* in the *REPEATED* statement, is used to calculate the “-2 Log Likelihood" for the covariance structure under *K*_*d*_.

*PROC MIXED* calculates “-2 Log Likelihood" under the heading of “Fit Statistics", see Appendix B. The above test statistic −2 ln*Λ*_*d*_ under *K*_*d*_ follows a chi-square distribution with degrees of freedom (d.f.) *ν*_*d*_, where *ν*_*d*_ is computed as

$$\begin{array}{@{}rcl@{}} \qquad \nu_{d} = \text{LRT df (under} H_{d}) - \text{LRT df (under} K_{d}). \end{array} $$

*PROC MIXED* calculates “LRT df" under the heading of “Null Model Likelihood Ratio Test", see Appendix B.

### Testing of hypothesis of difference in within-subject variabilities of KVX and CBCT

We test the difference between the repeatability coefficients of the two methods by testing the following hypothesis:

$$\begin{array}{@{}rcl@{}} H_{\sigma}:&& \text{the two methods do not have the same} \\&&\text{within-subject variabilities},  \\ \text{vs.} \;\;\;\; K_{\sigma}:&& \text{the two methods have the same} \\&&\text{within-subject variabilities},  \end{array} $$

As before here also we apply the likelihood ratio test for this hypothesis testing, and maximize the log likelihood function under both null hypothesis and alternating hypothesis separately to compute the test statistic −2 ln*Λ*_*σ*_, where

$$\begin{array}{@{}rcl@{}} \qquad \; \; -2 \ln \Lambda_{\sigma} =\left[-2 \ln \max_{K_{\sigma}} L \right] - \left[-2 \ln \max_{H_{\sigma}} L\right]. \end{array} $$

The option *TYPE=UN* in the *RANDOM* statement, along with *TYPE=UN* in the *REPEATED* statement, is used to calculate the “-2 Log Likelihood" for the covariance structure under *H*_*σ*_. *TYPE=UN* in the *RANDOM* statement, along with *TYPE=CS* in the *REPEATED* statement, is used to calculate the “-2 Log Likelihood" for the covariance structure under *K*_*σ*_. The test statistic −2 ln*Λ*_*σ*_ under *K*_*σ*_ follows a chi-square distribution with d.f. *ν*_*σ*_= LRT df (under*H*_*σ*_)−LRT df (under*K*_*σ*_).

### Testing of hypothesis of difference in overall variabilities of KVX and CBCT

We are interested in testing the following hypothesis:

$$\begin{array}{@{}rcl@{}} H_{\omega}:&& \text{the two methods do not have the same} \\&&\text{ overall variabilities},  \\ \text{vs.} \;\;\;\; K_{\omega}:&& \text{the two methods have the same} \\&&\text{ overall variabilities},  \end{array} $$

As before here also we apply the likelihood ratio test to compute the test statistic −2 ln*Λ*_*ω*_, where

$$\begin{array}{@{}rcl@{}} \qquad \; \; -2 \ln \Lambda_{\omega} =\left[-2 \ln \max_{K_{\omega}} L \right] - \left[-2 \ln \max_{H_{\omega}} L\right]. \end{array} $$

The option *TYPE=UN* in the *RANDOM* statement, along with *TYPE=UN* in the *REPEATED* statement, is used to calculate the “-2 Log Likelihood" for the covariance structure under *H*_*ω*_. The option *TYPE=CS* in the *RANDOM* statement, along with *TYPE=CS* in the *REPEATED* statement, is used to calculate the “-2 Log Likelihood" for the covariance structure under *K*_*ω*_. The test statistic −2 ln*Λ*_*ω*_ under *K*_*ω*_ follows a chi-square distribution with d.f. *ν*_*ω*_= LRT df (under*H*_*ω*_)−LRT df (under*K*_*ω*_).

## Results

Selected parts of the SAS output to test the within-subject variabilities are given in Appendix B. We present the sample SAS code (see Appendix A) to test within-subject variabilities by fitting the linear mixed effects model to our KVX and CBCT shifts for the lateral (X). We see that

$$\begin{array}{@{}rcl@{}} -2 \ln \max_{K_{\sigma}} L = 273.7 \quad \text{and} \quad -2 \ln \max_{H_{\sigma}} L = 239.7, \end{array} $$

with

$$\begin{array}{@{}rcl@{}} \text{LRT df (under} H_{\sigma}) = 5 \quad \text{and} \quad \text{LRT df (under} K_{\sigma})= 4. \end{array} $$

Therefore,

$$\begin{array}{@{}rcl@{}} -2 \ln \Lambda_{\sigma} &=&\left[-2 \ln \max_{K_{\sigma}} L \right] - \left[-2 \ln \max_{H_{\sigma}} L\right]\\&=& 273.7 - 239.7 = 34.0, \end{array} $$

with

$$\begin{array}{@{}rcl@{}} \nu_{\sigma} \!\,=\, \text{LRT df (under} H_{\sigma}) - \text{LRT df (under} K_{\sigma})= 5-4=1. \end{array} $$

The p-value for testing the within-subject variabilities of the two methods by using IML procedure of SAS is calculated at the third stage (see Appendix A). The *p* − value= 5.5112*E* − 9 (see Appendix B).

Inter-method bias, inter-method agreement, intra-method agreement, overall agreement and correlation results from COM_3_PARE are presented in Tables 1, 2, 3, 4 and 5. Using COM_3_PARE, in this specific head and neck positional verification demonstration dataset, while inter-method bias was <1 mm for all axes, a statistically significant between method bias was noted in the Z-axis (superoinferior axis). Also, was evidenced there was a statistically significant difference between CBCT and KVX inter-subject variation in the Z-axis (Table 1). Intra-subject variability was noted to be statistically significant for X- and Z-axes, as was overall variation. Correlation coefficient calculation estimation was performed using a mixed effects model (as per Roy [52]).

**Table 1:** Between-method bias

	Bias (cm)	*p* − value
X	0.0335	0.6077
Y	-0.0428	0.2836
Z	-0.0942	0.0253

**Table 2:** Inter-method agreement

	KVX (cm)	CBCT (cm)	*p* − value
X	0.0413	0.0670	0.4795
Y	0.0511	0.0464	0.7518
Z	0.0273	0.0848	0.0010

**Table 3:** Intra-method agreement

	KVX (cm)	CBCT (cm)	*p* − value
X	0.3396	0.1047	5.5 ×10^−9^
Y	0.1687	0.1693	1.0
Z	0.0485	0.0825	0.0034

**Table 4:** Overall agreement

	KVX (cm)	CBCT (cm)	*p* − value
X	0.3809	0.1717	3.7 ×10^−8^
Y	0.2198	0.2157	0.9512
Z	0.0758	0.1673	1.3 ×10^−5^

**Table 5:** Mixed effects estimated correlation coefficient

	Correlation
	coefficient
X	0.5329
Y	0.8038
Z	0.7336

Using the aforementioned criteria, automated shifts from CBCT and kV X-ray, acquired and processed in the manner detailed are interchangeable only for measurements of the Y-axis (anteroposterior), and for example, should not be used on alternating days in facilities with both systems in either X- or Z-axis. Additionally, our method suggests that, with lower intra-method variability in the X-axis (lateral), CBCT is the preferred measurement method, while in the Z-axis (superoinferior) kV X-ray measurement is preferable.

## Discussion

The necessity for quantitative evaluation of competing measurement devices, in cases where on device has not been found to be superior, is a significant need in science generally [1, 2, 5–7], and particularly within the radiological sciences community. Specifically, this issue is encountered when comparing distinct positional verification methods for image-guided radiotherapy [34, 53–56]. The difficulty of assessing competing platforms is particularly vexing, as it impedes efforts at cross platform comparison. Our group [24, 50] and others have implemented several distinct methods for presenting such analysis [25, 27, 33, 34, 45, 57]. Our previous efforts have utilized several extant method comparison statistical presentations (including Bland-Altman^7^, Lin’s concordance [58], Deming orthogonal regression [59, 60]); however, what was gained in completeness was lacking in parsimony. To this end, we sought to define an improved algorithm for practical comparison of distinct imaging methodologies, with a non-fixed number of repeated measurements per patient, in the absence of a “gold standard”. Often, inappropriate statistical analyses are implemented in lieu of formal method comparison statistics. The analysis of different measurement devices is not as straightforward as the initial observer may suppose. Bland and Altman demonstrated that mean comparison and linear regression are insufficient for comparison of differing measurement techniques [1]. The Bland- Altman method is succinct and easily interpretable, making it a classic of medical literature. In a series of seminal papers [1–7], Bland and Altman defined the standard methodology for comparing differing measurements, as well as establishing effective techniques accounting for inter- and intra-method variability/repeatability. However, while the Bland- Altman methodology remains the current benchmark, it fails (by design, one should note) to include generation of a formalized *p* − value, instead recommending that a clinically meaningful difference between measures be utilized. Additionally, though repeatability estimation is a recommended component of accurate method comparison, the calculation for greater than two replicates is somewhat unwieldy using the methodology proposed by Bland and Altman. Since many IGRT datasets span > 30 repeated daily measures, the utility of a statistical methodology which can readily integrate large replicate numbers is desirable. The COM_3_PARE methodology presented herein represents an attempt to integrate several desirable methodological attributes into a unified, readily performed statistical process. COM_3_PARE has several advantages over existing method comparison statistical analyses. Specifically, compared to general linear model [61, 62] (GLM)-based approaches (such as the *t*-test, linear regression, and ANOVA [63]), which fail to account for multiple sources of random variance, the linear mixed effects (LME)-based COM_3_PARE platform integrates variation estimation at multiple hierarchical levels (i.e., between- and within- measurement methods/subjects) [48]. From a practical point of view, this allows factor-wise assessment of procedural or technical variability of each of the two methods rather than a combined assessment, so that there is the capacity to determine the exact source of disagreement. COM_3_PARE is also resilient with regard to uneven numbers of replicates per device, a feature of great practical utility in a clinical setting, such as daily IGRT recording, where the number of IGRT fractions received for each patient may differ based on fractionation regimen of clinical exigency. Additionally, since COM_3_PARE has the capacity to fit differences in said variability to a hypothesis testing-friendly Bonferroni-corrected *p* − value output, while still implementing clinician-determined thresholds for agreement there is greater interpretability of statistical output, with no loss of clinical relevance. For instance, one could specify a priori that measurement differentials >1 mm would represent a lack of interchangeability globally. Data presentation was performed in this study in an effort to illustrate potential applications of COM_3_PARE for replicated image-based measurements of the kind frequently encountered in radiation oncology. The specific dataset included have been previously presented using standard method approaches. By revisiting these data using compare we hope to illustrate implementation of what we perceive to be a more usable and parsimonious approach to conceptualizing method comparison for IGRT applications, expanding upon, rather than obviating the previous work. With regard to the specific dataset presented herein, our analysis points to the difficulties possible when comparing IGRT platforms. For instance, having set our criteria pre-analysis, we were surprised to note that differing measurement methods proved preferable in distinct axes (e.g., CBCT in X-axis, kV X-ray for the Z-axis), while appearing by said criteria interchangeable in the Y-axis. A possible explanation of this phenomenon may appear as a feature of the imaging methodologies themselves. For CBCT, before three-dimensional reconstruction, data is acquired as axial slices (X-axis), while, previous to DRR referencing, the kV X-ray system uses orthogonal projections at oblique angles, parallel to the superoinferior plane (Z-axis). Consequently, method intra-subject repeatability may be tied to the reference plane of image acquisition, though this remains conjecture based on a single dataset. To our knowledge this technique represents the first formal hypothesis testing approach to integrate inter-method bias, inter-subject variability, and intra-subject variability of two methods with any number of replicated measurements for image-guided radiotherapy. As modeled on the aforementioned conceptual schema presented in the “Methods” section, we postulate that the following criteria be formally evaluated as feature of future image-guided radiotherapy measurement comparison studies comparing two imaging platforms, where multiple repeated observations on the same subject is possible. To meet our criteria for interchangeability [48]:

1. The bias and overall agreement must fall within a pre-specified range (e.g., bias/agreement of <0.1 cm between IGRT devices).
2. There should be no statistically significant, using a pre-specified threshold (e.g., <0.05) difference in the inter-subject variability of the two methods.
3. There should be no statistically significant difference in the intra-subject variability (i.e., repeatability) of the two methods.
4. In cases where criteria 2 and 3 are NOT met, the preferred IGRT technique is the one exhibiting the lower intra-subject variability (i.e., greater repeatability).

**Fig. 1:**
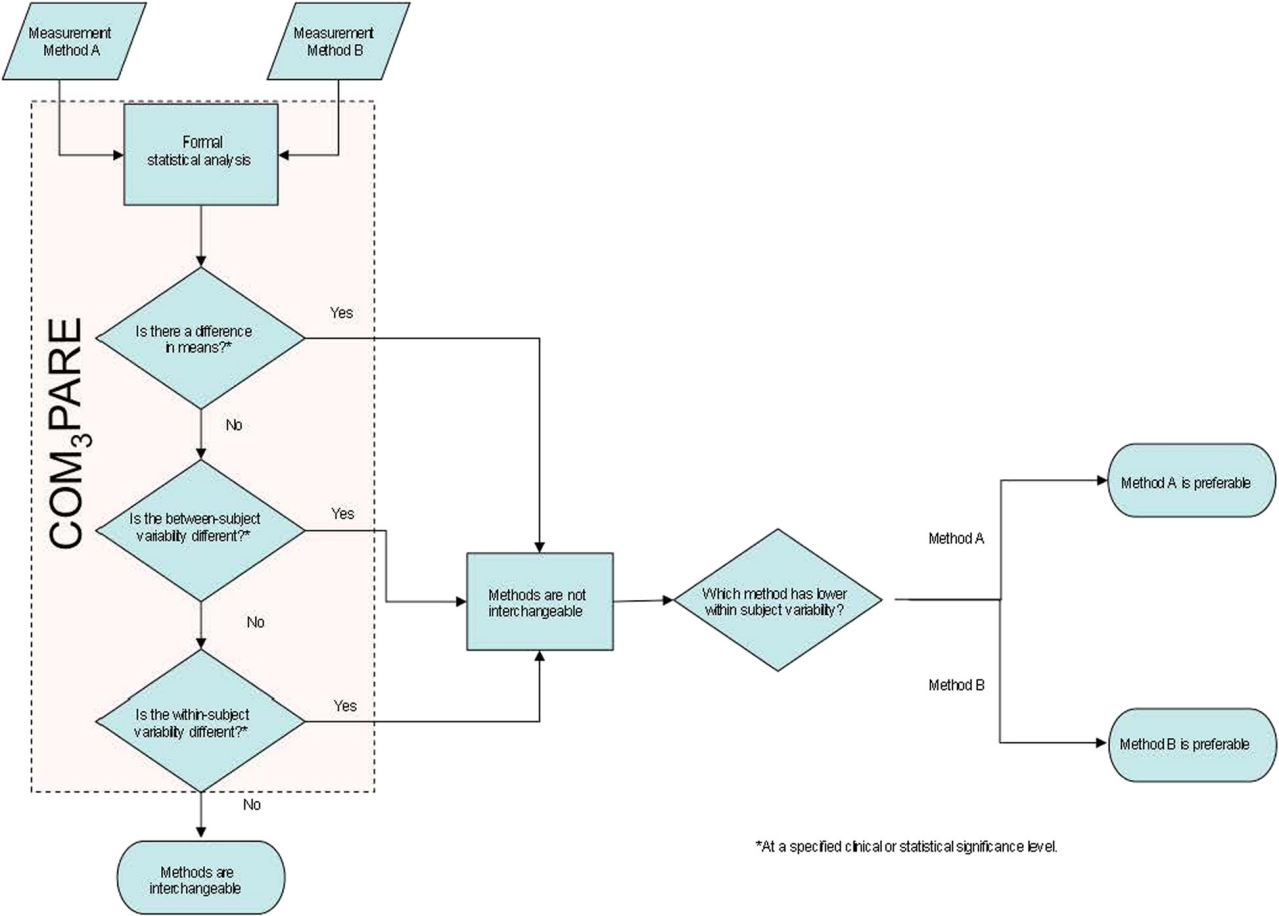
Flowchart of COM_3_PARE schema for IGRT device comparison

These criteria are presented as a graphical schema (Fig. 1); notably analysis of criteria 1–3 is easily incorporated in a single step using the COM_3_PARE SAS Code (Appendix A). The a priori criteria set we specified for interchangeability represented what we considered a reasonable metrics for the given application (i.e. fractionated radiotherapy of 30+ fractions for head and neck cancer) with a standardized PTV margin. The COM3PARE methodology, however, allows specification of any specified difference/p-value combination. Consequently, if a scenario arose whereby either tighter tolerances are desirable (e.g. 3-fraction SBRT), such parameters can be easily defined as an acceptability criteria.

## Conclusion

COM_3_PARE represents an attempt at a unified conceptual schema and analytic algorithm for method compassion of IGRT platforms. Initial application in a head and neck positional verification dataset shows feasibility and utility.

## Appendix A

### SAS code

Below we provide the sample SAS code to test within-subject variabilities by fitting the linear mixed effects model to our KVX and CBCT shifts for the lateral (X). We first fit the linear mixed effects model for the null hypothesis, then we fit the linear mixed effects model for the alternating hypothesis, and then find the *p* − value for the test. Appropriate changes can be made to test between-subject variabilities and overall variabilities using the SAS commands as described in Sections Testing of hypothesis of difference in between-subject variabilities of KVX and CBCT and Testing of hypothesis of difference in overall variabilities of KVX and CBCT. Appropriate changes can be also made for vertical (Y) and longitudinal (Z) dimensions and for any other data sets.





## Appendix B

### SAS output for covariance structure under the null and the alternating hypotheses

Below we provide the selected portions of the output of the above program.




